# Heterogeneity of MSC: Origin, Molecular Identities, and Functionality

**DOI:** 10.1155/2019/9281520

**Published:** 2019-07-02

**Authors:** Xueqing Liu, Tao Rui, Sicai Zhang, Zhaoping Ding

**Affiliations:** ^1^Department of Cardiology, Danyang People's Hospital, West Xinmin Rd. 5, 212300 Danyang, China; ^2^Departments of Medicine, Pathology and Laboratory Medicine, Schulich School of Medicine and Dentistry, Western University, London, Ontario, Canada N6A 4G5; ^3^Department of Microbiology and Immunobiology, Harvard Medical School, Department of Urology, Boston Children's Hospital, EN1070, 300 Longwood Ave., Boston, MA 02115, USA; ^4^Department of Anesthesiology, Changhai Hospital, Navy Medical University, Changhai Rd. 168, 200433 Shanghai, China; ^5^Institute of Molecular Cardiology, Heinrich-Heine University of Düsseldorf, Moorenstr. 5, 40225 Düsseldorf, Germany

“All animals are equal, but some animals are more equal than others.”

Animal Farm by George Orwell.

Mesenchymal stromal/stem cells (MSCs) are naturally one of the most abundant subsets of stromal cells within near all types of tissues over the body. In terms of biological definition and surface identities, MSCs were in general proposed by the International Society for Cellular Therapy (ISCT) with positive expression of CD73, CD90, and CD105, while lacking the surface marker of CD11b, CD14, CD19, CD34, CD45, CD79a, and HLA-DR. However, the recognition of the heterogeneity within the MSC pool leads to a large body of comparative studies that demonstrate MSCs may function differentially in the scenario of tissue repair [1]. However, the diversity of MSCs in the context of embryonic origins, anatomic locations, and biological properties is often ignored, as MSCs are broadly assumed with similar epitope identities and differentiation potentials, which implies that multiple tissues are equally suitable cell sources for the regeneration of multiple tissues [2]. Recently, most attention in the domain of regenerative medicine is directed toward the discovery of individual MSC population in close proximity to their host tissue that likely share the similar developmental origin during organogenesis [3]. Many studies have now revealed that MSCs are largely tissue-committed progenitors and may display additional tissue-specific expression of surface antigens that categorize MSC into subpopulations that link not only to the differentiation potential into certain cell lineages but also to their regenerative capacity. Furthermore, MSCs even within the same tissue may vary dynamically in response to local inflammatory milieus after tissue damage [4] and subject to changes in biological functionality after in vitro isolation and expansion [5], which may also be involved in altered secretion of regulatory cytokines and growth factors. Therefore, elucidation of the heterogeneity MSC pool is of particular interest and significance in the field of cell-based therapy by utilizing optimal donner cells to maximize therapeutical benefits. The aim of this special issue is thus to define tissue-specific subsets of MSC that preferentially contribute to regenerating certain types of tissues according to their distinct secreting profile of trophic factors and modulatory property of local immune response.

In the current special issue, 12 articles, after careful evaluation and reviewing processes coordinated by our guest-editor team, were finally accepted for publication. Overall, 7 papers are dealing with the isolation and characterization of organ-specific MSCs. (1) E. Wolmarans et al. introduced a methodological update of side population (SP) assay and compared the diversity of SP derived from hematopoietic stem cells, adipose-derived stromal cells, and dental pulp. (2) From human epidural fat tissue, N. Al-Jezani et al. derived stem/progenitor cells and a subset of a *Prx1*-positive population in mouse epidural fat that appeared to contribute to the formation of dura of the spinal cord by lineage-tracing technique, suggesting that the epidural adipose MSCs may play a significant biological role within the local environment beyond a merely mechanical tissue buffer. A further study demonstrating the reparative activity of those cells after spinal cord injury requires more intricate conducing. (3) R. I. Dmitrieva et al. found that myogenic stem cells in skeletal niche are a mixed population that coexpress both MSC and myogenic markers, and the functional properties were preserved in heart failure patients, although their metabolic statue was altered. (4) B. L. Han et al. characterized peritoneal dialysis effluent-derived MSC that meets all necessary criteria of MSCs, suggesting an additional cell source of MSCs particularly in dialytic patients. (5) S. Wang et al. isolated MSCs from the human neonatal thymus collected during cardiac surgery and further demonstrated the therapeutic benefits after cardiac transplantation in a rat model of myocardial infarction. (6) I. Uzieliene et al. reviewed the biological properties and therapeutical potential of human menstrual blood-derived MSCs in the field of chondrogenic differentiation potential and suitability for application in cartilage regeneration, as menstrual MSCs have many advantages: easily and frequently accessible sample source and greater proliferation and differentiation potential as compared to bone marrow-derived MSCs. (7) R. Chen et al., by using a small molecular method, generated iPSC from genetically labeled iPSC (distinct from lentiviral labeling), which opens a new window of lineage tracing in the analysis of functionality after in vivo transplantation.

To the point of functional relevance, Q. Zhuang et al. overview the antifibrotic activity of MSCs. In the process of renal fibrosis, MSCs are found to almost participate throughout the process of renal fibrotic; thus, targeting MSCs represent a safe and efficient intervention in the treatment of renal fibrosis. K. Tsuji et al. further outlined the possible mechanism linked to MSC-induced protection against renal injuries and diseases. Through a large body of secretomes, including soluble factors, extracellular vesicles, and microRNA, MSCs are able to suppress cell apoptosis, cell necrosis, renal fibrosis, renal inflammation, and oxidative stress as well as to promote autophagy, but the effectiveness varies among MSCs derived from adipose tissue, bone marrow, and cord blood, suggesting therapeutic heterogeneity within the MSC pool. The diversity of MSCs was further demonstrated by three papers. (1) V. T. Nguyen et al. collected MSCs from bone marrow, from acetabular subchondral bone, and from trabecular bone on the femoral head with focus on osteogenic differentiation and found that the progenitor cells obtained from diverse surgical sites in a hip replacement procedure share common characteristics of MSC but differ in plasticity: in osteogenic differentiation condition, the cells from the acetabulum had the lowest accumulation of calcium deposit while the cells that originated from bone marrow and femur created a considerably increased amount of the deposit; in chondrogenic and adipogenic conditions, bone marrow cells possessed a predominant differential capacity compared with the others. (2) In a comparative study, W. Zarychta-Wiśniewska et al. revealed that more than 1400 genes were differentially expressed in MSCs derived from human bone marrow and adipose tissue, and all factors are associated with tenogenesis and chemotaxis but less age-related. (3) Notably, the study by K. Tan et al. nicely underscores the potential heterogeneity of MSCs derived from the same source (pericardial tissue)—based on the intensity of CD73 expression that is generally considered as a surface marker of MSCs. In this paper, they demonstrated that CD73 through its catalytic products (extracellular adenosine) is capable of modulating local immune response in the injured hearts and thus dictates the reparative properties.

In summary, these 12 outstanding original research articles and reviews have importantly touched the biological nature of MSCs, not only in animals but also in human beings. The understanding of the heterogeneity of the MSC pool may promote the development of novel and important innovations that enhance our natural ability to replace injured parts through the activation of the stem cell pool, which is often seen in amphibians and newborn mammalians.
